# Sex-related differences in the associations between diurnal cortisol pattern and social and emotional loneliness in older adults

**DOI:** 10.3389/fpsyg.2023.1199405

**Published:** 2023-09-07

**Authors:** María del Carmen Díaz-Mardomingo, Lucía Utrera, Shishir Baliyan, Sara García-Herranz, Juan Carlos Suárez-Falcón, Raquel Rodríguez-Fernández, Patricia Sampedro-Piquero, Azucena Valencia, César Venero

**Affiliations:** ^1^Department of Basic Psychology I, UNED, Madrid, Spain; ^2^Instituto Mixto de Investigación – Escuela Nacional de Sanidad (IMIENS), Madrid, Spain; ^3^Department of Psychobiology, UNED, Madrid, Spain; ^4^Escuela Internacional de Doctorado – Universidad Nacional de Educación a Distancia (EIDUNED), Madrid, Spain; ^5^Departamento de Psicología Experimental, Procesos Cognitivos y Logopedia, Instituto Pluridisciplinar, Universidad Complutense de Madrid, Madrid, Spain; ^6^Department of Basic Psychology II, UNED, Madrid, Spain; ^7^Department of Behavioral Sciences Methodology, UNED, Madrid, Spain; ^8^Department of Biological and Health Psychology, Universidad Autónoma de Madrid, Madrid, Spain

**Keywords:** aging, HPA axis, saliva, social loneliness, emotional loneliness, sex

## Abstract

**Introduction:**

Loneliness is a distressful feeling that can affect mental and physical health, particularly among older adults. Cortisol, the primary hormone of the Hypothalamic-Pituitary-Adrenal axis (HPA-axis), may act as a biological transducer through which loneliness affects health. While most previous studies have evaluated the association between loneliness, as a unidimensional construct, and diurnal cortisol pattern, no research has examined this relationship discriminating between social and emotional loneliness in older adults. As sex differences in the negative mental health outcomes of loneliness have been reported, we also investigated whether diurnal cortisol indices and loneliness associations occur in a sex-specific manner.

**Methods:**

We analyzed the diurnal cortisol- pattern in 142 community-dwelling, non-depressed, Caucasian older adults (55,6% female) aged 60-90. Social and emotional (family and romantic) loneliness scores were assessed using the Spanish version of the Social and Emotional Loneliness Scale for Adults (SELSA). Five salivary cortisol samples were used to capture key features of the diurnal cortisol pattern, including: awakening and bedtime cortisol levels, awakening response (CAR), post-awakening cortisol output (post-awakening cortisol [i.e., the area under the curve with reference to the ground: AUC_G_]), total diurnal cortisol release (AUC_G_), and diurnal cortisol slope (DCS).

**Results:**

After controlling for sociodemographic variables, the hierarchical linear multiple regression analyses revealed that in male older adults, higher scores on social and family loneliness were associated with elevated awakening cortisol levels, total diurnal cortisol output, and a steeper diurnal cortisol slope (DCS). However, these associations were not observed in female older adults. In addition, feelings of romantic loneliness were positively associated with bedtime cortisol levels and AUC_G_ in older males. Multilevel growth curve modeling showed that experiencing more social and emotional loneliness predicted higher diurnal cortisol output throughout the day in older male adults.

**Discussion:**

The presence of sex differences in the relationship between cortisol indices and loneliness among older adults holds particular significance for diagnostic and screening procedures. Combining loneliness scales as screening tools with diurnal cortisol measures has the potential to be an effective and cost-efficient approach in identifying higher-risk individuals at early stages.

## 1. Introduction

Loneliness is a psychological phenomenon that arises from the subjective perception of unfulfilled intimate and social relationship needs, leading to distressing feelings (Peplau and Perlman, 1982; Ernst and Cacioppo, 1999). Over the last decade, loneliness has become a significant public health concern due to its association with poor physical and mental health. As such, loneliness has been found to be a major risk factor for morbidity and premature mortality, particularly among older adults (Steptoe et al., 2013; Holt-Lunstad et al., 2015; Elovainio et al., 2017; Cacioppo and Cacioppo, 2018; Rico-Uribe et al., 2018; Schutter et al., 2021). The prevalence of loneliness varies from 5 to 34% being adolescents and older adults the most susceptible to suffer it (Steptoe et al., 2013; Qualter et al., 2015; Beutel et al., 2017).

Loneliness has been studied from both, uni-and multi-dimensional, conceptual perspectives. While some authors have considered loneliness as a unidimensional construct that fluctuates basically in intensity, but not in nature, others have proposed two types of loneliness based on the kinds of unmet needs; social loneliness and emotional loneliness (Weiss, 1973). Social loneliness results from the individual’s perception of not being part of an engaging community, whereas emotional loneliness arises from the absence of close emotional ties with someone who truly cares for and understands the individual (i.e., a spouse/partner, kin, or a best friend). Subsequently, other authors proposed that emotional loneliness is further comprised of two specific domains; family and romantic, affording greater precision in loneliness assessment (DiTommaso and Spinner, 1993).

Age and life-changing events that frequently occur later in life, such as deteriorating health and loss of a spouse/partner and/or friends, can differently account for the onset of emotional and social loneliness (Carstensen, 1992; De Jong Gierveld et al., 2015; Fierloos et al., 2021). Although feelings of loneliness can be experienced despite having frequent contact or even living with other people, living alone is a risk factor for feeling alone (Heinrich and Gullone, 2006). Additionally, older adults with lower educational level are more likely to acknowledge experiencing increased social and emotional loneliness (Cohen-Mansfield et al., 2016; Dahlberg et al., 2018; Fierloos et al., 2021).

Research on sex differences in loneliness has yielded inconclusive results. Several reports suggest that females experience greater loneliness than males (Pinquart and Sörensen, 2001; Aartsen and Jylhä, 2011; Cohen-Mansfield et al., 2016; Dong and Chen, 2017; Hyland et al., 2019; Fierloos et al., 2021), whereas other studies report a similar probability of occurrence in both sexes (DiTommaso and Spinner, 1993; Cramer and Neyedley, 1998; Steptoe et al., 2004; Leitch et al., 2018; Lee et al., 2019) or even a higher incidence in males (Dykstra and de Jong Gierveld, 2004; De Jong Gierveld and Van Tilburg, 2010; Djukanović et al., 2015; van den Broek, 2017; Theeke et al., 2019).

Loneliness is a psychosocial distressing feeling (Hawkley and Cacioppo, 2010; Miller, 2011; Quadt et al., 2020) that has been postulated to be associated with a dysfunction of the HPA axis (Steptoe et al., 2004; Doane and Adam, 2010; Cacioppo et al., 2015). The HPA axis is a crucial neuroendocrine system involved in the physiological stress response (Ulrich-Lai and Herman, 2009). The release of cortisol, the main end product of the HPA axis, fluctuates with a circadian rhythm, with a rapid increase in the first 30–45 min after waking (the cortisol awakening response: CAR) followed by a decline throughout the rest of the day (Pruessner et al., 1997). Various cortisol indices such as CAR, the diurnal cortisol slope (DCS) (i.e., the difference between morning and bedtime cortisol levels), and total cortisol released throughout the day (AUC_G_) represent distinct aspects of the basal diurnal cortisol pattern and may provide valuable and discrete measures associated with emotional well-being (Adam and Kumari, 2009; Herbert, 2013). However, the literature concerning the association between loneliness and the diurnal cortisol pattern has yielded inconsistent findings. While some studies reported that lonely individuals displayed a greater CAR compared to non-lonely adults (Steptoe et al., 2004; Adam et al., 2006; Doane and Adam, 2010), others found no changes in CAR (Schutter et al., 2017; Montoliu et al., 2019) or even a blunted CAR (Lai et al., 2018). Furthermore, certain studies indicated a flattener DCS (Johar et al., 2020) or higher diurnal cortisol secretion (Cacioppo et al., 2000; Lai et al., 2018), while others found no association between loneliness and DCS (Schutter et al., 2017; Montoliu et al., 2019) or diurnal cortisol output (Rueggeberg et al., 2012; Montoliu et al., 2019).

Distinguishing between social and emotional (romantic or family) loneliness domains can be relevant in older adults as feeling social loneliness is qualitatively distinct from emotional loneliness (DiTommaso and Spinner, 1993; Weiss, 1998; Peerenboom et al., 2015). Social loneliness is often associated with exclusion, boredom, passivity, aimlessness, and depression, whereas emotional loneliness is frequently related to feelings of anxiety, insecurity, and desolation (Weiss, 1973; Creecy et al., 1985; Larson, 1990). However, to the best of our knowledge, no previous studies have examined whether there are variations in the relationship between diurnal cortisol indices and loneliness based on the specific type of loneliness (social or emotional) among older adults. This aspect remains unexplored and warrants further investigation to gain a comprehensive understanding of the association between different forms of loneliness and cortisol patterns in this population. We postulate that social and emotional loneliness may be associated with specific diurnal cortisol patterns, reflecting an adaptation of the HPA axis. As loneliness has been linked to depression (Alpass and Neville, 2003; Hawkley and Cacioppo, 2003; Domènech-Abella et al., 2017) and individuals with depression often exhibit altered cortisol patterns (Pruessner M. et al., 2003; Stetler and Miller, 2011; Belvederi Murri et al., 2014; Rhebergen et al., 2015), we excluded participants with major or probable major depression from our study. This exclusion aimed to capture the association between loneliness and circadian cortisol levels before depression could potentially influence them. By doing so, we aimed to obtain a clearer understanding of the relationship between loneliness and cortisol patterns among our study participants.

In this study, we conducted hierarchical linear multiple regression analyses to examine the relationship between various diurnal cortisol indices (awakening cortisol levels, bedtime cortisol levels, CAR, post-awakening cortisol AUCG, total diurnal cortisol release [AUCG], and diurnal cortisol slope [DCS]) and social or emotional loneliness dimensions in community-dwelling, non-depressed older adults. Drawing on previous research that highlighted distinct associations between social and emotional loneliness and health problems, with emotional loneliness being more prevalent and health-damaging than social loneliness (Peerenboom et al., 2015; O’Súilleabháin et al., 2019), we hypothesized that emotional loneliness would exhibit a stronger association with an altered diurnal cortisol pattern compared to social loneliness. Due to the mixed findings in the literature regarding the link between loneliness and diurnal cortisol indices, we were unable to definitively determine the direction of these associations. However, considering reported evidence showing stronger associations between feelings of loneliness and adverse mental health outcomes, such as depression, low life satisfaction, and resilience, in older males compared to older females (Holwerda et al., 2012; Zebhauser et al., 2014; De Jong Gierveld et al., 2015), and the indication of altered diurnal cortisol levels in adult and older males experiencing loneliness (Papp et al., 2013; Johar et al., 2020), we anticipated that the relationship between social and emotional loneliness with diurnal cortisol indices would be more pronounced in older males than in females.

The present study aimed to achieve two main objectives. Firstly, we aimed to explore the potential association between emotional and social loneliness and diurnal cortisol patterns in older adults. Secondly, we sought to investigate whether this association displays a stronger effect in males compared to females.

## 2. Materials and methods

### 2.1. Participants

As part of a broader investigation, we initially recruited 212 Caucasian participants through an advertisement placed in cultural and educational centers across several municipalities of the Community of Madrid. The participants did not receive any monetary or economic compensation for their involvement in the study. Their participation was entirely voluntary, driven by their interest in contributing to scientific research and their curiosity about the topic under investigation. Participants were recruited between spring and winter, when saliva cortisol levels exhibit peak values (Miller et al., 2016). The study’s exclusion criteria were as follows: (a) presence of neurodegenerative or endocrine disease; (b) presence of disabling chronic disease; (c) diagnosed psychiatric disorder; (d) suspicion of depression based on a GDS-15 score higher than 5; (e) diabetes; (f) lack of independence in daily activities; (g) history of alcohol or drug abuse and; (h) use of any medication known to influence cortisol levels, such as corticosteroid-based medications or opioids, as previously reported (Nicolson, 2008).

After taking into consideration the exclusion criteria, the initial sample of participants was reduced to 165. Subsequently, 23 subjects did not collect the five salivary samples and/or did it at different time points of the day than requested and were unable to repeat the sampling procedure. As a result, the final sample was composed of 142 older adults, ranging from 60 to 90 years old (*M* = 67.72, *SD* = 5.70).

### 2.2. Procedure

All procedures complied with specifications outlined by the Communities Council Directive 2001/20/EC Declaration of Helsinki, and the Ethics Committee at the Universidad Nacional de Educación a Distancia (UNED) approved the study. All participants received verbal and written information about the study and provided written consent.

Subjects were interviewed to collect personal information and sociodemographic data, as well as information on their lifestyle and habits. The interviews with participants were conducted by a team of specialized psychologists. The neuropsychological assessment involved the application of the Spanish version (Martinez de la Iglesia et al., 2005) of the short form of the Geriatric Depression Scale (Yesavage and Sheikh, 1986) to assess the participant’s emotional state. Given that altered HPA-axis function is frequently observed in depressed patients (Belvederi Murri et al., 2014), we excluded participants with a GDS score higher than 5.

### 2.3. Social and emotional loneliness

To evaluate social and emotional (romantic and family) loneliness in older adults, we used the Spanish version (Yárnoz-Yaben, 2008) of the short form of the Social and Emotional Loneliness Scale for Adults (SELSA-S) (DiTommaso et al., 2004). The SELSA-S is a multidimensional measure of loneliness that comprises 15 items rated on a 7-point Likert-type scale, ranging from 1 (strongly disagree) to 7 (strongly agree). It measures emotional (romantic and family) and social loneliness. The SELSA-S’s three subscales are a valid measure of loneliness (DiTommaso et al., 2004; Çeçen, 2007). Each subscale consists of five statements about feelings of loneliness within the past year. The family loneliness subscale assesses feelings toward family relationships. The social loneliness subscale measures feelings concerning belonging to a social group. The romantic loneliness subscale assesses the degree to which participants feel they have significant others in their lives. Mean scores are calculated for each subscale, and higher SELSA-S scores indicate higher levels of loneliness in the particular domain. In the current study, the estimated reliability values for each of the three SELSA-S subscales calculated using Cronbach’s alpha were: *α*_family loneliness_ = 0.78, *α*_social loneliness_ = 0.79, and *α*_romantic loneliness_ = 0.68.

### 2.4. Salivary sampling and assay protocol

Salivary collection protocol was explained to each study participant and they were shown the correct use of the Salivette salivary collection device (Sarstedt, Nümbrecht, Germany) by a trained research associate. Participants were told not to eat, drink, smoke, brush their teeth, or use mouthwash 30 min before salivary collection. Subjects collected saliva on weekdays using the provided cotton swabs for 1 min. In the morning three saliva samples were collected (immediately upon awakening, at 0.5 h, and 0.75 h after waking), to assess the CAR, and the post-awakening cortisol secretion (post-awakening AUC_G_), using the formulae indicated by Pruessner J. C. et al. (2003). In addition, two more samples were collected in the afternoon (7 h after waking) and at bedtime. Diurnal cortisol slope (DCS) was calculated by subtracting cortisol measured at awakening from cortisol measured at bedtime and dividing this by the total hours between the two sample collection points. Thus, lower (more negative) slopes indicate a more rapid decline in cortisol levels, whereas slope values closer to zero reflect flatter diurnal rhythms. Total cortisol secretion over the day was estimated using the area under the curve with respect to ground (AUC_G_) defined by all cortisol data points across the day (Pruessner J. C. et al., 2003). Participants were told of the importance of accurate timing of the salivary collections and were asked to keep a log of their real sampling times even if deviations from the requested procedure occurred. Participants were instructed to collect and store the samples in their freezer until we collected them within the following few days. Subsequently, samples were stored at−80°C until they were analyzed. Saliva cortisol levels were determined in duplicate in our lab using a commercially available enzyme immunoassay kit (Salimetrics, State College, PA). The integrated optical density for each sample was determined at a wavelength of 450 nm using a Microplate Reader (DigiScan Reader V3.0 and DigiWIN software; ASYS Hitech GmbH, Austria). The plates were read within 10 min of adding the stop solution. Intra- and inter-assay precision of 3.5 and 5.2% respectively, and an assay sensitivity of 0.03 ng/mL.

### 2.5. Statistical analysis

The basic descriptive analyzes were performed for the variables sex, age, years of education, marital status, living status (living alone vs. with others), depression (GDS), and measures of loneliness (SELSA-S). Next, Student’s *t*-test and chi-square analyses, when appropriate, were used to investigate sex differences in the sociodemographic variables and the three subtypes of loneliness. Significant deviations from normality were detected in cortisol values, so values were subjected to a log transformation. Extreme values ±3 SD from the mean were identified, and z scores were winsorized.

The relationship between each subtype of loneliness and the cortisol indices was analyzed using hierarchical multiple linear regression analyses. Independent analyses were performed for each cortisol index (awakening cortisol levels, bedtime cortisol levels, CAR, post-awakening AUC_G_, AUC_G_, and DCS) as the response variable. For unadjusted analyses, each type of loneliness was included in step one. For adjusted analyses, age, partner status, years of formal education, and living alone were retained as covariates in step one, and each subtype of loneliness in step two. These covariates are frequently included in loneliness studies and have been independently associated with loneliness among older adults (Cohen-Mansfield et al., 2016; Fierloos et al., 2021). Subsequently, we analyzed whether there were sex differences in the association between each subtype of loneliness and cortisol indices by repeating these analyses and including the covariates, each subtype of loneliness and sex in step one, and the interaction loneliness * sex in step two.

Finally, we used multilevel modeling to examine the associations of each type of loneliness with the diurnal cortisol pattern of each participant (Raudenbush and Bryk, 2002). This statistical procedure accounts for the non-independence of observations and allows the evaluation of within and between-person predictors of diurnal cortisol parameters (Adam et al., 2006). The five diurnal cortisol samples of each participant were Ln transformed prior to the full information maximum likelihood (FIML) procedure to estimate the parameters of the models. To determine the best-fitting curve for the data, we used linear and quadratic growth curve models.

To perform these statistical analyses, version 25.0 of SPSS was used. The moderation analyses in hierarchical multiple linear regression analyses were conducted by PROCESS macro for SPSS version 3.4.^1^ All *p* values were two-tailed, and the level of significance was taken as *p* < 0.05.

## 3. Results

Our sample was composed of 142 Caucasian older adults (63 males and 79 females) and sociodemographic characteristics of the participants are described using percentages, or mean (standard deviation, SD) when appropriate, as a function of sex (see Table 1). Males and females did not significantly differ in age (*p* = 0.456) or depression score (*p* = 0.471), but there were significant differences in years of formal education, partner status, and living alone (all *p* < 0.001). Romantic loneliness scores were higher in females than in males (*U* = 1,698; *p =* 0.001), but no significant differences were observed in social and family loneliness ratings between both sexes (*p* = 0.535 and *p* = 0.328, respectively).

**Table 1:** Demographic characteristics of the study sample.

	Total (*N* = 142)	Men (*N* = 63)	Women (*N* = 79)	
Sex (%)		44.4	55.6	
				*P*
Age M (SD)	67.72 (5.70)	67.32 (6.18)	68.04 (5.30)	0.209
Years of education (SD)	14.76 (5.04)	16.41 (4.92)	13.44 (4.76)	0.001
Marital status (%)				0.001
Married	67.6	30.0	37.6	
Single	9.9	4.4	5.5	
Divorced	10.6	4.7	5.9	
Widowed	12.0	5.3	6.7	
Living status (%)				0.001
Living alone	28.9	12.8	16.1	
Living with others	71.1	31.6	39.5	
Depression (GDS)	1.61 (1.53)	1.51 (1.47)	1.70 (1.59)	0.522
Social loneliness	10.11 (5.24)	10.00 (4.61)	10.19 (5.72)	0.649
Family loneliness	8.68 (5.64)	8.43 (5.08)	8.89 (6.07)	0.788
Romantic loneliness	16.19 (9.01)	13.74 (8.44)	18.10 (9.09)	0.007

Significant *p*-values are in bold type.

⁎ using chi-square statistics for categorical variables, Student’s *t*-tests for continuous variables and Mann–Whitney *U*-tests in case of non-normal distributions.

Initially, we performed Pearson’s correlation analysis between the diurnal cortisol indexes and each type of loneliness. Social loneliness was positively related to awakening cortisol levels (*r_s_* = 0.18, *p = 0*.033) and post-awakening AUC_G_ (*r_s_* = 0.19, *p = 0*.029), and negatively related to cortisol slope (*r_s_* = −0.17, *p = 0*.044). Family loneliness was positively related to awakening cortisol levels (*r_s_* = 0.18, *p = 0*.030) and romantic loneliness did not correlate to any of the cortisol indexes (all *p* < 0.440). In addition, a Spearman correlation analysis indicated that age was correlated to social (*r_s_* = 0.26, *p = 0*.002), but not romantic (*r_s_* = 0.08, *p = 0*.358) or family (*r* = 0.04, *p = 0*.671) loneliness. Living alone was positively related to romantic (*r_s_* = 0.63, *p* < 0.001), but not to family or social loneliness (all *p* > 0.432). Years of education were marginally related to family (*r_s_* = 0.15, *p = 0*.071), but not to romantic or social loneliness (all *p* > 0.435).

### 3.1. Association between diurnal cortisol indexes and social loneliness

Multiple linear regression estimates for the association of diurnal cortisol indexes and each subtype of loneliness are summarized in Table 2. Unadjusted analyses did not show significant associations between social loneliness scores and any cortisol measures or indices. After adjusted analyses of covariates (age, years of formal education, partner status, and living status-living alone *vs* living with others), positive significant associations were observed between social loneliness and waking cortisol (*β* = 0.175, *p < 0.*05), as well as post-awakening AUC_G_ (*β* = 0.185, *p < 0*.05). A negative association was observed between social loneliness and DCS (*β* = −0.217, *p < 0*.05). After inclusion of covariates, sex, and social loneliness in step one and social loneliness * sex interaction in step 2, a significant interaction effect was observed in waking cortisol (*β* = 0.689, *p < 0*.01), post-awakening cortisol AUC_G_ (*β* = 0.484, *p < 0*.05), AUC_G_ (*β* = 0.499, *p < 0*.05) and DCS (*β* = −0.626, *p < 0*.01). In all the (loneliness * sex) significant interactions, the conditional effects of the moderator sex were significant for males. No significant interactions were observed between social loneliness and sex for any of the other studied cortisol indices in either unadjusted or adjusted analyses (all *p* ≥ 0.218) (see Table 2).

**Table 2:** Regression analyses with social loneliness or social loneliness*sex as predictors, and the cortisol indexes as dependent variables, unadjusted and adjusted for covariates.

	Unadjusted analyses Social loneliness			Adjusted analyses		
	*R*^2^ change	Beta	*p* value	*R*^2^ change	Beta	*p* value
Waking cortisol	0.018	0.135	0.110	0.057	0.050	0.175
Bedtime cortisol	0.007	0.086	0.312	0.043	0.049	0.585
CAR	0.000	0.011	0.903	0.022	−0.018	0.845
Post-awakening_AUCG_	0.027	0.166	0.052	0.084	0.185	0.038
Total cortisol AUC_G_	0.010	0.101	0.243	0.042	0.100	0.277
DCS	0.024	−0.156	0.065	0.079	−0.217	0.014

	Unadjusted analyses Social loneliness* sex		Adjusted analyses			
	*R*^2^ change	Beta	*p* value	*R*^2^ change	Beta	*p* value
Waking cortisol	0.112	0.685	0.000	0.140	0.689	0.000
Bedtime cortisol	0.019	0.249	0.218	0.049	0.188	0.356
CAR	0.010	−0.052	0.805	0.026	−0.073	0.730
Post-awakening_AUCG_	0.112	0.494	0.013	0.135	0.484	0.016
Total cortisol AUC_G_	0.093	0.515	0.012	0.103	0.499	0.017
DCS	0.106	−0.604	0.002	0.149	−0.626	0.001

The table shows the standardized beta coefficients.

### 3.2. Association between diurnal cortisol indexes and family loneliness

Unadjusted and adjusted regression analyses showed a significant positive association between family loneliness and waking cortisol levels (*β* = 0.213, *p < 0.*05 and *β* = 0.209, *p < 0*.05) and a negative between family loneliness and DCS (*β* = −0.219, *p < 0.*01 and *β* = −0.212, *p < 0*.05). None of the other associations between family loneliness and cortisol indexes were statistically significant (all *p* ≥ 0.07). After adjusted covariates, a significant interaction of family loneliness * sex was also observed in waking cortisol levels (*β* = 0.428, *p < 0*.01), post-awakening AUC_G_ (*β* = 0.617, *p < 0*.01), total cortisol release during the day (AUC_G_) (*β* = 0.567, *p < 0*.01) and DCS (*β* = −0.415, *p < 0*.05), with a significant conditional effect of the moderator sex for males, but not for females (see Table 3).

**Table 3:** Regression analyses with family loneliness or family loneliness*sex as predictors, and the cortisol indexes as dependent variables, unadjusted and adjusted for covariates.

	Unadjusted analyses Family loneliness			Adjusted analyses		
	*R*^2^ change	Beta	*p* value	*R*^2^ change	Beta	*p* value
Waking cortisol	0.045	0.213	0.011	0.073	0.209	0.013
Bedtime cortisol	0.023	0.153	0.070	0.060	0.140	0.098
CAR	0.007	−0.082	0.341	0.029	−0.089	0.302
Post-awakening_AUCG_	0.022	0.149	0.082	0.071	0.133	0.117
Total cortisol AUC_G_	0.008	0.087	0.314	0.037	0.068	0.439
DCS	0.048	−0.219	0.009	0.082	−0.212	0.012

	Unadjusted analyses Family loneliness * sex			Adjusted analyses		
	*R*^2^ change	Beta	*p* value	*R*^2^ change	Beta	*p* value
Waking cortisol	0.100	0.403	0.012	0.121	0.428	0.009
Bedtime cortisol	0.043	0.269	0.104	0.073	0.229	0.172
CAR	0.038	0.205	0.224	0.042	0.206	0.236
CAR_AUCg_	0.162	0.599	0.000	0.183	0.617	0.000
AUCg cortisol	0.122	0.577	0.000	0.138	0.567	0.001
DCS	0.105	−0.385	0.017	0.129	−0.415	0.012

The table shows the standardized beta coefficients.

### 3.3. Association between diurnal cortisol indices and romantic loneliness

Unadjusted and adjusted regression analyses showed no significant association between romantic loneliness scores and any of the cortisol indices. A significant interaction of romantic loneliness * sex was related to bedtime cortisol levels (*β* = 0.431, *p < 0*.05 and *β* = 0.434, *p < 0*.05, for unadjusted and adjusted analyses, respectively) and cortisol AUC_G_ in unadjusted (*β* = 0.513, *p < 0*.01) and adjusted analyses (*β* = 0.513, *p < 0*.01). There were no significant interactions between romantic loneliness and sex for any of the other studied cortisol indexes in either unadjusted or adjusted analyses (see Table 4).

**Table 4:** Regression analyses with romantic loneliness or romantic loneliness*sex as predictors, and the cortisol indexes as dependent variables, unadjusted and adjusted for covariates.

	Unadjusted analyses Romantic loneliness			Adjusted analyses		
	*R*^2^ change	Beta	*p* value	*R*^2^ change	Beta	*p* value
Waking cortisol	0.001	0.033	0.698	0.049	0.180	0.103
Bedtime cortisol	0.010	0.101	0.232	0.051	0.131	0.235
CAR	0.000	−0.022	0.801	0.022	0.033	0.770
Post-awakening_AUCG_	0.000	0.004	0.959	0.065	0.144	0.200
Total cortisol AUC_G_	0.002	0.047	0.592	0.040	0.114	0.322
DCS	0.001	−0.035	0.678	0.060	−0.195	0.076

	Unadjusted analyses Romantic loneliness * sex			Adjusted analyses		
	*R*^2^ change	Beta	*p* value	*R*^2^ change	Beta	*p* value
Waking cortisol	0.028	0.266	0.130	0.062	0.229	0.198
Bedtime cortisol	0.055	0.431	0.014	0.093	0.434	0.015
CAR	0.014	0.140	0.432	0.030	0.126	0.489
Post-awakening_AUCG_	0.071	0.328	0.060	0.102	0.298	0.091
Total cortisol AUC_G_	0.108	0.513	0.003	0.123	0.513	0.004
DCS	0.022	−0.100	0.573	0.063	−0.058	0.747

The table shows the standardized beta coefficients.

### 3.4. Multilevel modeling of diurnal cortisol patterns as a function of each type of loneliness

Since most of the diurnal variation in cortisol levels is explained by time of day (Adam et al., 2006), we used multilevel modeling procedures to test whether the pattern of diurnal cortisol levels differed as a function of each type of loneliness. In this study, a two-level multilevel growth-curve analysis was applied. In level-1 participants’ cortisol values were predicted by different indicators of cortisol variation throughout the day. After adjusting different models, we observed that the quadratic growth curve best fitted to predict diurnal cortisol variation, only results from the quadratic term were interpreted and described. The intercept was set to the cortisol level at waking, time after waking was used as the time metric, and CAR was coded as a dummy variable, in which the sample of cortisol level at 30 min was assigned a value of 1, and the other samples were set to 0.

The final growth curve model fitted contained these parameters: the intercept (${\;}_{\neq 0i}$), which is the waking value of subject *i* (in Ln (μg/dL); the coefficient for CAR (${\;}_{\neq 1i}$) reflected the change in cortisol between the waking and 30-min post-awakening cortisol samples measurement *i*; ${\;}_{\neq 2i}$ and ${\;}_{\neq 3i}$ reflected, respectively, the linear time (initial slope immediately after waking (in units of Ln(μg/dL) per hour or the instantaneous growth rate for subject *i* at time waking) and the quadratic time changes in cortisol (quadratic slope -rate of deceleration- or the curvature or deceleration in each growth trajectory of each participant); and $e_{\text{ti}}$ was the residual term. A presentation of the level-1 model equation of cortisol activity is thus:


$${\text{LNCORT}}_{\text{ti}}=\pi_{0i}+\pi_{1i}\text{CAR}+\pi_{2i}\text{Time}+\pi_{3i}{\text{Time}}^{2}+e_{\text{ti}}$$

Because it has been fitted to a multilevel LNcortisol unconditional quadratic growth model (random-coefficient regression model), the previous equation specifies the level-1 model and the level-2 was:


$$\pi_{0i}=\beta_{00}+u_{0i}$$

$$\pi_{2i}=\beta_{20}+u_{2i}$$

where *β*_00_ = mean LNcortisol at waking, *β*_20_ = mean LNcortisol growth rate at waking, u_0i_ and u_2i_ are the random effects (variance components) at level-2.

The results showed that the estimations of the four fixed effects parameters were significant: Intercept (${\hat{\pi }}_{0i}$ = −1.078, SE = 0.037, *p* < 0.01), CAR (${\hat{\pi }}_{1i}$ = 0.236, SE = 0.040, *p* < 0.01), Time (${\hat{\pi }}_{2i}$ = −0.209, SE = 0.011, *p* < 0.01) and Time^2^ (${\hat{\pi }}_{3i}$ = 0.005, SE = 0.001, *p* < 0.01). The model that best fits to predict the daily cortisol pattern of the participants is a quadratic growth model with the inclusion of a CAR parameter for the peak in the cortisol sample at minute 30. On the other hand, if we look at the intercept and time (slope) of random effects, we see that the variance of both parameters is significant [*Var*(*u*_0*i*_) = .103, Z = 5.602, *p* <.01 *and Var*(*u*_2*i*_) = .002, Z = 5.386, *p* < .01], indicating that there is variability among the participants, so it is advisable to introduce predictor variables of level-2 to explain this observed variability. The CAR and Time^2^ parameters of random effects (variance components) did not show significant variability among the participants or had convergence problems when these parameters were estimated, and there were not included in the level-2 model.

In the level-2 model, the sociodemographic variables (age, education, partner status, living alone, and sex) and types of loneliness were entered as predictors of variability parameters significant to level-1. We carried out different models to predict the variability in participants’ parameters, intercept and time. The best fit and more parsimonious model was one in which the variables predicted the variation in the intercept parameter, but not in the time parameter. Therefore, the final fully adjusted cortisol model combining levels 1 and 2 for each type of loneliness was:


$$\begin{array}{l} {\text{LNCORT}}_{\text{ti}}=\beta_{00}+\beta_{01}\text{Age}+\beta_{02}\text{Education}+\beta_{03}\text{Partner Status}\\ \;+\beta_{04}\text{Living Alone}+\beta_{05}\text{Sex}+\beta_{06}\text{Loneliness}\\ \;+\beta_{07}\text{Sex}*\text{Loneliness}+\beta_{10}\text{CAR}+\beta_{20}\text{Time}\\ \;+\beta_{30}{\text{Time}}^{2}+u_{0i}+u_{2i}\text{Time}+e_{\text{ti}} \end{array}$$

In Table 5, we present the results of the multilevel model for predicting diurnal cortisol patterns as a function of the different types of loneliness: family, romantic and social, moderating by sex, controlling for age, educational level, partner status, and living status (alone or with others).

**Table 5:** Multilevel model results for predicting diurnal cortisol pattern as a function of subtype of loneliness, moderating by sex, controlling for age, educational level, marital status, and living alone or with others.

Parameter	Fixed effects					
	Family loneliness		Romantic loneliness		Social loneliness	
	*β*	SE	*β*	SE	*β*	SE
Intercept ($\beta_{00}$)	−1.578483**	0.410952	−1.703227**	0.445353	−1.447266**	0.423262
CAR ($\beta_{10}$)	0.236882**	0.039903	0.236011**	0.039900	0.236147**	0.039909
Time ($\beta_{20}$)	−0.209103**	0.010864	−0.209013**	0.010864	−0.208922**	0.010866
Time^2^ ($\beta_{30}$)	0.004886**	0.000630	0.004880**	0.000630	0.004876**	0.000630
Age	0.005262	0.005108	0.006774	0.005171	0.002799	0.005354
Educational level	0.004987	0.006094	0.009371	0.006107	0.008679	0.006089
Marital status	−0.016418	0.039469	−0.015349	0.040121	−0.023100	0.040907
Living alone/with others	0.054616	0.073117	0.061517	0.089336	0.035766	0.073811
Subtype of loneliness	−0.003395	0.005768	−0.004337	0.005252	−0.000138	0.006643
Sex	−0.192663	0.111909	−0.198812	0.124771	−0.213736	0.136110
Subtype of loneliness × sex	0.033336**	0.009863	0.018909**	0.006553	0.030257**	0.011467

Parameter	Random effects					
	Family loneliness		Romantic loneliness		Social loneliness	
	Variance	Wald Z	Variance	Wald Z	Variance	Wald Z
Intercept ($u_{0i}$)	0.083929**	5.162	0.091295**	5.335	0.086718**	5.231
Time ($u_{2i}$)	0.001511**	5.385	0.001512**	5.387	0.0.01509**	5.382
Residual error ($e_{\text{ti}}$)	0.150508		0.150468		0.150547	

**p < 0.01.

**Figure 1:**
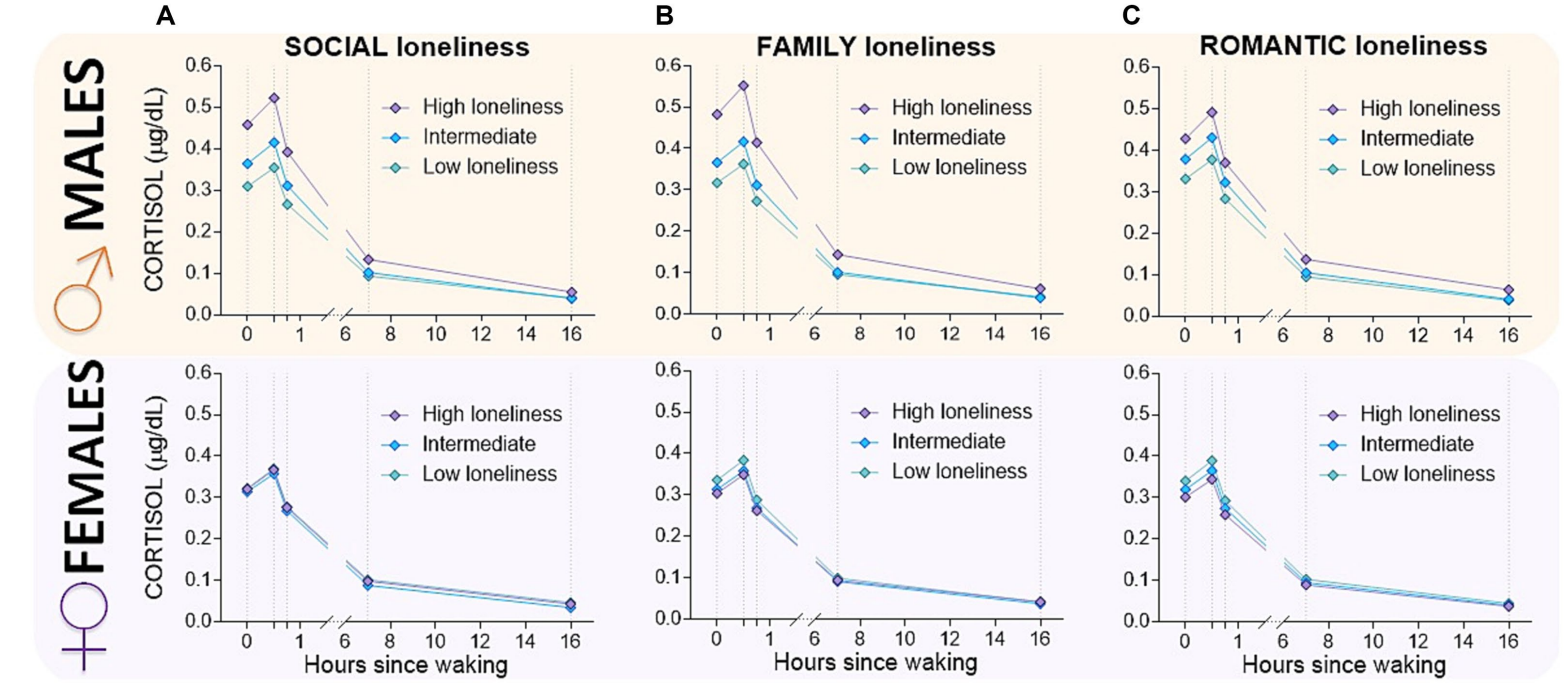
Multilevel growth curve predicted mean cortisol levels throughout the day of older males and females by quartile groups of **(A)** Social loneliness; **(B)** Family Loneliness and **(C)** Romantic loneliness. To establish the groups, we considered quartiles 1 (Q_1_) and 3 (Q_3_) and the interquartile range (from Q_1_ to Q_3_). Thus, the lower group comprised participants with scores below Q_1_; the middle group was composed of participants within the interquartile range (from Q_1_ to Q_3_); and, finally, the higher group comprised participants with scores above Q_3_. The logarithmic cortisol values were reconverted to the original metric to facilitate understanding of the graphs.

The between-person associations of each type of loneliness (i.e., family, romantic and social) with the quadratic cortisol curves were highly significant for Intercept, CAR, Time, and Time^2^ (all *p* < 0.001), after adjusting for age, educational levels, partner status, living status, and sex. In addition, a significant interaction effect was found for sex and: (i) family loneliness (β= 0.033; *p* < 0.01); (ii) romantic loneliness (β= 0.019; *p* < 0.01), and (iii) social loneliness (β= 0.030; *p* < 0.01). Likewise, after considering all the predictors and covariates, the intercept and time variability remain significant for the three types of loneliness, although an approximate 2% reduction in the intercept’s variance is observed when the predictors of level 2 were included in the model, being more prominent in family loneliness (Figure 1).

## 4. Discussion

Our investigation revealed that social and emotional loneliness feelings are associated with diurnal cortisol indices in male, but not female, older adults. The results indicate that greater social and emotional loneliness, specifically in the family and romantic domains, were linked to increased total diurnal cortisol output (AUC_G_). Family and social loneliness were positively associated with higher cortisol levels upon waking and a steeper DCS. Although CAR was not associated with any type of loneliness, higher post-awakening cortisol AUC_G_ index was positively related to greater social loneliness in older males. Furthermore, romantic loneliness scores were associated with bedtime cortisol levels. Additionally, multilevel growth curve modeling revealed that scores in each type of loneliness strongly predicted individual differences in diurnal cortisol patterns in male but not female older adults.

In our sample, social and family loneliness scores were similar in older females and males. However, higher levels of romantic loneliness were found in older females compared to males, which might be partially explained by the unequal distribution of risk factors such as partner status, as well as living alone *vs* with others or educational level (Dykstra and Fokkema, 2007; Belvederi Murri et al., 2014). Thus, previous studies have indicated that whereas having an intimate partner, such as a spouse, is a protective factor against romantic loneliness (Dykstra and Fokkema, 2007; Aartsen and Jylhä, 2011; Fierloos et al., 2021), the lack or loss of a romantic partner is a risk factor for emotional loneliness in both, males and females, and for social loneliness in males (Dykstra and de Jong Gierveld, 2004; Dykstra and Fokkema, 2007; Drennan et al., 2008). Additionally, a low educational level is a risk factor for loneliness, probably because it could lead to lesser prospects for social participation and reduced social networks (Routasalo and Pitkala, 2003; Dykstra and de Jong Gierveld, 2004).

Our data revealed that family and social loneliness scores were positively associated with elevated cortisol levels at awakening, post-awakening cortisol AUC_G_, total diurnal cortisol output (AUC_G_), and steeper DCS in older males, but not in females, after adjusting for different covariates that can affect loneliness and/or cortisol levels such as age, years of education, partner status (widowed/divorced/single vs married/partnered), and living status (living alone vs with others). The literature exploring the association between loneliness and cortisol indices in older adults has yielded inconsistent results. Thus, using the *University of California, Los Angeles’ Loneliness Scale* (UCLA), a unidimensional loneliness instrument (Russell et al., 1978), some studies found no relationship between loneliness scores and total cortisol output (AUC_G_) in older adults (Rueggeberg et al., 2012; Montoliu et al., 2019), whereas others reported increased salivary cortisol levels across the course of a day in chronically lonely adults (Cacioppo et al., 2000). Further, our results indicating no association between any type of loneliness and DCS in female older adults are in line with several studies in older adults (Steptoe et al., 2004; Adam et al., 2006; Schutter et al., 2017; Montoliu et al., 2019), but differ from Johar et al. (2020) who observed that loneliness was associated with a flattered DCS. In addition, we found no association between CAR and either social or emotional loneliness scores, similar to previous findings (Montoliu et al., 2019), but in contrast to other studies reporting increased CAR (Adam et al., 2006; Doane and Adam, 2010). Interestingly, here we observed that romantic loneliness scores were positively related to bedtime cortisol levels in older males, but not in females. In a previous study, Montoliu et al. (2019) reported that loneliness was associated with bedtime cortisol levels in older adults, although they observed no sex differences. However, there is inconsistency in the literature regarding this relationship. Thus, some authors reported that loneliness feelings in adults could predict higher CAR levels on the following day, but not on the same day (Adam et al., 2006; Doane and Adam, 2010), whereas others indicated significantly diminished CAR in recently lonely married older males, but not females, compared to not-lonely counterparts (Johar et al., 2020) or reduced post-awakening cortisol output in lonely and severely lonely older adults (Schutter et al., 2017).

Study differences in the instruments used for loneliness assessment and sociodemographic characteristics of the sample (including the age of participants, the proportion of males and females, partner status, and inclusion/exclusion criteria concerning mental and physical health) may account for some discrepant findings between our present study and previous research. Here, we used the SELSA-S to assess both social and emotional loneliness, encompassing both family and romantic aspects. This methodological approach distinguishes our research from most previous studies in older adults, where loneliness was typically measured as a single construct using the UCLA loneliness scale. The only exception was a study by Schutter et al. (2017), who used the De Jong Gierveld Loneliness to assess both social and emotional loneliness (de Jong-Gierveld and Kamphuls, 1985). However, feeling lonely in a certain domain of loneliness (i.e., social loneliness) can be qualitatively distinct from feeling lonely in another (i.e., emotional loneliness) (DiTommaso et al., 2004). Despite being related, both social and emotional loneliness are considered distinct constructs (Green et al., 2001; DiTommaso et al., 2005; De Jong Gierveld and Van Tilburg, 2010; Fierloos et al., 2021) and several factor analytic studies have reported that research instruments that discriminate between social and emotional loneliness are more appropriate to assess loneliness than those using a unidimensional scale (DiTommaso and Spinner, 1993; Cramer and Barry, 1999; De Jong Gierveld and Van Tilburg, 2010; Liu and Rook, 2013).

Besides the instrument used to assess loneliness, the characteristics of the sample may also explain some differences between our present results and previous studies concerning the relationship between loneliness and diurnal cortisol indices. Thus, while we excluded depressed participants from the study sample, other studies included participants with major depression (Steptoe et al., 2004; Doane and Adam, 2010; Schutter et al., 2017; Johar et al., 2020). Although feeling lonely does not necessarily involve being clinically depressed (Perissinotto et al., 2012), loneliness feelings have been reported to precede the onset of depression (Cacioppo et al., 2006; Cheng et al., 2008), a negative mood that has been related to affect cortisol patterns (Pruessner M. et al., 2003; Stetler and Miller, 2011; Belvederi Murri et al., 2014; Rhebergen et al., 2015).

In our study, the positive association between social and emotional loneliness scores and cortisol output throughout the day in male older adults may reflect the allostatic load on the HPA axis. Loneliness is considered a distressful feeling that may elicit the response of central and peripheral pathways that can also affect HPA activity and cortisol levels (Kirschbaum and Hellhammer, 1989; Johnson et al., 1992). Several epidemiological studies have provided evidence that control of HPA activity worsens with aging, possibly reflecting the wear and tear in biological stress systems (Nater et al., 2013). Interestingly, increased cortisol release throughout the day has been reported to occur in older ages (Heaney et al., 2012; McEwen and Morrison, 2013; Nater et al., 2013), a phenomenon seen more markedly in males (Kumari et al., 2010; Karlamangla et al., 2013). As cortisol exerts a critical role in energy mobilization and consumption, the increase in morning cortisol levels has been speculated to prepare the brain for workload and cognitive challenges of the upcoming day (Schlotz et al., 2004; Adam et al., 2006; Fries et al., 2009; Stalder et al., 2010; Xiong et al., 2021). Moreover, diurnal cortisol indices have been proposed to be useful biomarkers of cortisol’s effects on brain structures involved in emotional processing (Rhebergen et al., 2015) and cortisol is thought to play an adaptive function in mobilizing the coping resources needed to appraise one’s current state of social connections and develop new ones (Del Giudice et al., 2011). Therefore, it may be postulated that the positive association between both, social and family loneliness and the diurnal cortisol indices observed in the present study may reflect the inputs of socioemotional experiences that are coordinated in different corticolimbic and associated brain structures that regulate the circadian activity of the HPA axis (Kirschbaum and Hellhammer, 1989; Johnson et al., 1992) and are involved in emotional processing (Rhebergen et al., 2015). Whether the observed cortisol indices associated with social and family loneliness scores in older males may be of potential useful prognostic capability for predicting the evolution from loneliness to depression or other mental or health problems is worthy of further study.

Our results reveal a differential association of social and emotional loneliness with HPA axis dynamics in older males and females. Several reasons may be postulated to explain the observed sex-specific differences in these associations, including socio-cultural and biological factors. Thus, several studies have indicated that older males exhibit stronger links between feelings of loneliness and adverse mental health outcomes, including depression, low life satisfaction, and resilience, when compared to older females (Holwerda et al., 2012; Zebhauser et al., 2014; De Jong Gierveld et al., 2015, but see also Richard et al., 2017). More recently, a study reported that loneliness in older adults was only associated with psychological health in males, but not in females (Crespo-Sanmiguel et al., 2022). Sex differences in the association between cortisol and loneliness may also be attributed to the use of emotion-focused coping strategies, which are reported to be more frequently used by females than males (Kelly et al., 2008). Emotion-focused coping strategies involve managing and regulating emotional experiences in response to stressors or challenging situations (Lazarus and Folkman, 1984). Research has indicated that adaptive emotion-focused coping, such as seeking social support or positive reappraisal can be linked to lower cortisol levels throughout the day (O’Donnell et al., 2008). Biological factors may also account for the sex differences in the associations between diurnal cortisol observed in the present study. Thus, the gradual and continuous decline in testosterone levels that occurs in males over 40 (Feldman et al., 2002) may also impact cortisol levels. Studies involving leuprolide administration in males, a drug that reduces testosterone release, have shown that testosterone replacement leads to a decrease in CRH-stimulated plasma cortisol levels. This suggests a suppressive influence of testosterone on cortisol levels, which aligns with findings from studies involving rodents (Rubinow et al., 2005). Additionally, higher levels of circulating cortisol-binding globulin (CBG) observed in older females compared to males (Kudielka et al., 2009) may act as a buffer for free cortisol levels.

Our study has some limitations to be considered. First, the findings reported here are cross-sectional and limit the conclusions that can be drawn. Evidence of a causal effect of loneliness on diurnal cortisol levels in older males requires longitudinal studies. Second, compliance of salivary cortisol samples with the study protocol was not done using an electronic device. However, we provided extensive advice to the participants for the salivary sampling procedure as previously recommended by Adam and Kumari (2009). Third, the possibility of false positives from multiple statistical testing may be of concern. However, the consistent nature of the associations found between social and emotional loneliness and the different cortisol indices supports the likelihood that statistically significant associations were not random.

In conclusion, this study used a convenience sample of healthy community-living older adults without depression to investigate the associations between social and emotional loneliness and cortisol indices. We found positive associations between social and emotional loneliness scores and post-awakening AUC_G_, and total cortisol output for males. These associations remained significant even after adjusting for age, years of education level, depressive score, partner status, and living status. The present study highlights the importance of adopting a multidimensional approach to loneliness when examining its relationship with diurnal cortisol levels in older males and females, and this bears significant relevance for diagnostic and screening procedures. Future research in this field has the potential to investigate the mediating role of the HPA axis in the sex-specific connections between loneliness and health conditions. Based on our findings, we recommend the integration of loneliness scales as screening tools with diurnal cortisol measures to identify higher-risk individuals at early stages within a large cohort. This approach can enhance the timely implementation of preventive interventions, optimizing their effectiveness.

## Data availability statement

The raw data supporting the conclusions of this article will be made available by the authors, without undue reservation.

## Ethics statement

The studies involving humans were approved by Ethics Committee at the Universidad Nacional de Educación a Distancia (UNED). The studies were conducted in accordance with the local legislation and institutional requirements. The participants provided their written informed consent to participate in this study.

## Author contributions

MD-M and CV: conceptualization. JS-F and RR-F: methodology and statistical analysis. LU, SB, SG-H, MD-M, and PS-P: data collection. LU, SB, SG-H, MD-M, AV, and CV: investigation. CV: writing-original draft preparation and project administration. SB, MD-M, PS-P, AV, and CV: writing-review and editing. All authors have read and approved the published version of the manuscript.

## Funding

This research was supported by RTI2018-094627-B-I00 and PID2021-125945OB-I00 (Spanish Ministry of Science and Innovation) and by the Cátedra de Igualdad y Bienestar Psicológico y Emocional (Diputació de Castelló-UNED).

## Conflict of interest

The authors declare that the research was conducted in the absence of any commercial or financial relationships that could be construed as a potential conflict of interest.

## Publisher’s note

All claims expressed in this article are solely those of the authors and do not necessarily represent those of their affiliated organizations, or those of the publisher, the editors and the reviewers. Any product that may be evaluated in this article, or claim that may be made by its manufacturer, is not guaranteed or endorsed by the publisher.

## Footnotes

^1^ www.processmacro.org
